# The graded novelty encoding task: Novelty gradually improves recognition of visual stimuli under incidental learning conditions

**DOI:** 10.3758/s13428-022-01891-8

**Published:** 2022-06-13

**Authors:** Richárd Reichardt, Bertalan Polner, Péter Simor

**Affiliations:** 1 Department of Cognitive Science, Budapest University of Technology and Economics, Budapest, Hungary; 2 Institute of Pedagogy and Psychology, Eötvös Loránd University, Szombathely, Hungary; 3 Institute of Psychology, Eötvös Loránd University, Budapest, Hungary; 4 UR2NF, Neuropsychology and Functional Neuroimaging Research Unit at CRCN - Center for Research in Cognition and Neurosciences and UNI - ULB Neurosciences Institute, Université Libre de Bruxelles (ULB), Brussels, Belgium

**Keywords:** Recognition memory, Novelty, Visual stimuli, Incidental learning, Parametric manipulation

## Abstract

It has been argued that novel compared to familiar stimuli are preferentially encoded into memory. Nevertheless, treating novelty as a categorical variable in experimental research is considered simplistic. We highlight the dimensional aspect of novelty and propose an experimental design that manipulates novelty continuously. We created the Graded Novelty Encoding Task (GNET), in which the difference between stimuli (i.e. novelty) is parametrically manipulated, paving the way for quantitative models of novelty processing. We designed an algorithm which generates visual stimuli by placing colored shapes in a grid. During the familiarization phase of the task, we repeatedly presented five pictures to the participants. In a subsequent incidental learning phase, participants were asked to differentiate between the “familiars” and novel images that varied in the degree of difference to the familiarized pictures (i.e. novelty). Finally, participants completed a surprise recognition memory test, where the novel stimuli from the previous phase were interspersed with distractors with similar difference characteristics. We numerically expressed the differences between the stimuli to compute a dimensional indicator of novelty and assessed whether it predicted recognition memory performance. Based on previous studies showing the beneficial effect of novelty on memory formation, we hypothesized that the more novel a given picture was, the better subsequent recognition performance participants would demonstrate. Our hypothesis was confirmed: recognition performance was higher for more novel stimuli. The GNET captures the continuous nature of novelty, and it may be useful in future studies that examine the behavioral and neurocognitive aspects of novelty processing.

**Supplementary Information:**

The online version contains supplementary material available at 10.3758/s13428-022-01891-8.

## Introduction

Memory is highly selective, and the rules governing the entry of information into memory systems are a central concern of memory research (Baddeley et al., 2020; Eichenbaum, 2017). Among the many variables influencing memory formation, novelty is probably the most intimately connected with memory, since it is defined in terms of what is already represented in memory (Barto et al., 2013). Several researchers have put forth theories on why and how novelty plays a central role in memory formation (Barto et al., 2013; Duszkiewicz et al., 2019; Frank & Kafkas, 2021; Press et al., 2020; Quent et al., 2021; Reichardt et al., 2020; Schomaker & Meeter, 2015; Van Kesteren et al., 2012).

Early on, Tulving and colleagues formulated a theory on the beneficial effect of novelty on memory: they noticed that several brain regions, among them the hippocampus, responded differently to novel than to familiar stimuli (Tulving et al., 1996; Tulving & Kroll, 1995). This inspired the novelty/encoding hypothesis (Tulving & Kroll, 1995), which states that the hippocampal activation elicited by novel stimuli is due to a memory encoding process instantiated by novelty detection. According to this classic theory, memory performance should be better for novel items than for familiar ones due to this increased hippocampal activation.

However, the novelty/encoding hypothesis is too simplistic to describe the full breadth of findings related to the memory effects of novelty (Barto et al., 2013; Duszkiewicz et al., 2019; Kafkas & Montaldi, 2018; Press et al., 2020; Reichardt et al., 2020). More specifically, familiar stimuli are sometimes better remembered than novel ones (Poppenk et al., 2010), and neural responses to novel stimuli reduce dramatically over time (Murty et al., 2013; Nieuwenhuis et al., 2011). Furthermore, challenges emerged at the conceptual level as well, in that novelty can be assorted into different categories which may elicit different memory processes (Duszkiewicz et al., 2019; Frank & Kafkas, 2021; Quent et al., 2021; Reichardt et al., 2020). For example, Berlyne, in his pioneering studies of novelty and its effects on cognition, distinguished between complete and relative novelty (Berlyne, 1960). Complete novelty is attributed to a stimulus that the observer has never encountered before, while a stimulus with relative novelty may only be novel due to the unfamiliar combination of well-known features. More recently, authors differentiated between absolute and contextual novelty, although these categories are essentially the same as complete and relative novelty (Kafkas & Montaldi, 2018). Since Berlyne, many researchers have emphasized that novelty should be understood as a continuum which is theoretically crucial in that it allows us to study the dose–response effects of novelty on memory (Frank & Kafkas, 2021; Quent et al., 2021; Reichardt et al., 2020). And indeed, recent cognitive neuroscientific studies yielded convergent evidence showing that the degree of novelty has a major influence on memory formation (Duszkiewicz et al., 2019; Reichardt et al., 2020; Van Kesteren et al., 2012).

Accordingly, treating novelty as a categorical variable may not be appropriate, yet it is still often done in cognitive psychology, and most experimental paradigms do not capture gradual effects of novelty (Greve et al., 2017; Kumaran & Maguire, 2006, 2009). For instance, Greve and colleagues taught participants associations between scene categories and emotional words and assessed memory performance for novel congruent and incongruent pairings (Greve et al., 2017). In a sense, this manipulation generates two levels in overall novelty: congruent novelty is less novel, while incongruent novelty is more novel. They showed that incongruent novel pairings were recognized with a significantly higher probability. This finding is in line with the idea that the more a given episode differs from previously experienced episodes, the more likely it is to be remembered later (Van Kesteren et al., 2012). Nonetheless, the relationship between novelty and memory formation is more complicated. For example, it has been shown that low and high schema congruency are both likely to boost memory performance, that is, there is a U-shaped relationship between congruency and memory (Greve et al., 2019). Since schema congruency is directly related to novelty, the association between novelty and memory should also be assessed with a method that enables handling novelty as a continuous variable. In order to fill this gap in the available paradigms of cognitive neuroscience, we have developed the Graded Novelty Encoding Task that manipulates the degree of novelty in the stimulus set in a parametric manner, and hence, provides a tool to investigate the dose-dependent impact of novelty on memory processing.

Another important caveat with the behavioral methods currently used in cognitive neuroscience is that they handle novelty in an overly simplistic manner. In most cases, any stimulus is considered to be novel as long as it is presented for the first time during the experimental procedure. This is true for paradigms that use distinct stimuli as the source of novelty (stimulus novelty) and for paradigms that use spatial and temporal compositions (contextual and associative novelty). The manipulations in these paradigms theoretically fit the definition of novelty: any event that has not been encountered before is novel. However, since human memory is far from perfect, likely not everything that fits the definition of novelty theoretically can be considered novel for the experimental participants in practice. For example, participants viewed colored fractals in an experiment (Schomaker & Meeter, 2012). Importantly, the novel fractals were only presented once during the experiment; however, the participants viewed hundreds of these during the task. It is doubtful that all these stimuli can be considered novel by the nervous system. In support of this idea, there are several results that show that repetition of novel stimuli results in the gradual decrease of the brain responses associated with novelty detection (Murty et al., 2013; Nieuwenhuis et al., 2011). In our opinion, this problem also calls for continuous novelty manipulations: if the novel stimuli differ in the degree of theoretical novelty in a quantifiable manner, we can infer what degree of theoretical novelty elicits behavioral or neurophysiological phenomena suggestive of novelty detection. Thus, manipulating novelty in a continuous manner may reveal more about the neural background of novelty detection and the associated memory processes.

We developed an algorithm to produce stimuli between which the differences can be calculated and used this stimulus pool in a task where these differences define the degree of novelty of a stimulus. We named this task the Graded Novelty Encoding Task (GNET). In the GNET, participants are presented with simple, colored geometric shapes arranged in a 3x3 grid at the center of the screen. The images containing the colored shapes can be described by three matrices containing information on layout, shape, and color. This way, the features of the images are converted into numerical values and the differences between the stimuli can be easily quantified. By using a simple experimental design in which the participants are familiarized with a few stimuli and then try to distinguish these from novel stimuli, we can assess whether the differences between stimuli are meaningful, in the sense that they elicit measurable behavioral differences. Using the GNET, we replicated the classical finding that more novel items are more likely to be recognized on a memory test, and showed that the memory-enhancing effects of novelty are expressed in a graded manner.

## Methods

### Participants and procedure

We collected data from 140 university students reporting no prior or current history of psychiatric, neurological or chronic somatic disorders through an online interface. The participants were recruited at the Eötvös Loránd University and were compensated by partial credit points. To ensure data quality, we applied stringent exclusion criteria adapted to the online assessment (Grootswagers, 2020) and excluded participants who missed more than a third of the trials (*N* = 6), participants who were not able to memorize the familiar stimuli (*N* = 27) and participants who overused the response reflecting uncertainty in the last phase (*N* = 12) (see details under “Experimental design”). Therefore, we analyzed a sample of 95 participants (78 females, mean ± standard deviation age: 24.8 ± 6.9). The study was approved by the Hungarian Ethical Review Committee for Research in Psychology and participants provided online informed consents. The study was conducted in accordance with the Declaration of Helsinki.

Participants signed up using a Google Form that was followed by an automatic e-mail at the time they designated on the form as suitable for taking part in the experiment. The e-mail contained a link to another Google Form, which served as a registration interface and assessed additional variables focusing on the state (e.g. alcohol, nicotine, and caffeine consumption, sleep-wake schedules, etc.) of the participants prior to running the experimental task. These variables were used only to screen the state of participants to ensure data quality (e.g. serious sleep deprivation, substance use, psychiatric or neurological condition). We did not exclude participants based on this data from the final analysis. The task itself was run in the browser using JATOS [just another tool for online studies] (Lange et al., 2015). Most of the participants completed the task during the afternoon (14:00–18:00 CEST) and the experiment took approximately 20 minutes to complete.

We also conducted a laboratory experiment to verify that the task could be used in laboratory-based neuroimaging studies. We recruited 24 participants from the same university participant pool. We enrolled individuals who reported no prior or current history of psychiatric, neurological or chronic somatic disorders. The participants of the laboratory experiment completed the same task as the participants in the online study, using the same online interface from a computer in the laboratory. We excluded participants due to inattentiveness (*N* = 1) and the overuse of “maybe seen” responses (*N* = 4). This left a total of 19 participants in the final sample (15 females, mean ± standard deviation age: 19.6 ± 1.9).

### Experimental task

In order to investigate the graded effect of novelty on memory performance, we designed an incidental learning task with a recognition memory test.

#### Experimental design

The task consisted of three phases:

1. *Familiarization phase*: in a randomized order, five pictures (*“familiars”*) were shown for 3.0 seconds, five times each, with an interstimulus interval of 1.5 seconds. Participants were instructed to memorize these pictures.
2. *Incidental learning phase:* participants were presented with 75 pictures, each shown for 3.0 seconds, and participants had 3.0 seconds to respond. The intertrial interval (ITI) was 1.5 seconds. Participants were only instructed to indicate whether the picture they saw was one of the five familiars (*“old”* response, each familiar stimulus was presented five times each = 25 presentations) from the familiarization phase or a novel one (“*new”* response, 50 stimuli, each presented once), and were not told to memorize the pictures. As mentioned before, we excluded 27 participants, as the lower end of the confidence interval for the probability of success value of the binomial distribution describing the responses during this phase was below 0.33. The binomial distribution contained the correct (“*old”* response to familiar, “*new”* response to novel stimuli) and incorrect responses (“*new”* response to familiar, “*old”* response to novel stimuli) to the trials in the familiarization phase. The probability of success value would have been (*p* =) 0.33 if a participant responded with “*old”* on all trials, as the number of correct responses would have been 25 while the number of incorrect responses would have been 50 in this case. This exclusion criterion in our view left only those participants in the final analysis who could reliably distinguish the familiars from the novels over chance level.
3. *Recognition test:* the 50 novel pictures from the incidental learning phase and 50 distractor (previously not presented) pictures were shown. Each stimulus appeared for 3.0 seconds, and participants had 3.0 seconds to respond. The intertrial interval (ITI) was 1.5 seconds. Participants were asked to indicate with a button press whether they remembered seeing the picture in the incidental learning phase (*“already seen”* response) or not (*“not seen”* response). Participants could also respond *“maybe seen*,” if they were indecisive about the picture, but they were requested to only use this option as a last resort. As mentioned before, 12 participants were excluded due to high rates of *“maybe seen”* responses (> 25%), which suggested they did not follow the instructions.

The task was programmed with OpenSesame (Mathot et al., 2012).

#### Stimulus material

**Fig. 1:**
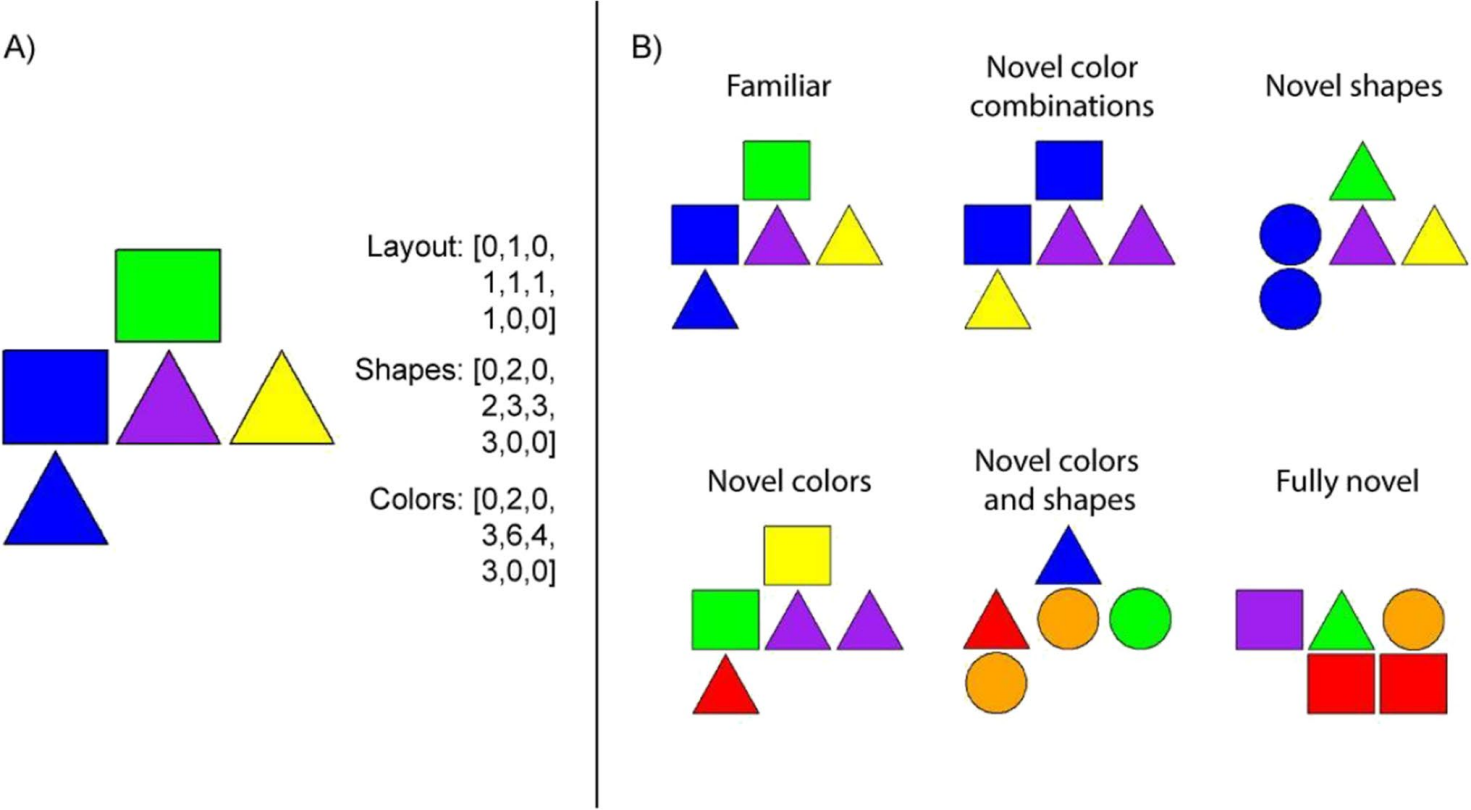
**a** The stimuli in the Graded Novelty Encoding Task can be described by three matrices representing layout, shapes and colors. The layout matrix describes which cells contain shapes in the 3x3 grid, the shapes matrix describes which shapes are in the cells of the grid (e.g. 2 – square; 3 – triangle) and the color matrix describes the color of the shapes in the grid (e.g. 3 – blue; 4 – yellow). These matrices can easily be compared and, thus, the difference between stimuli can be computed. **b** A familiar stimulus and examples of the different novelty categories. The first four categories use the same layout as the familiar that they are generated from. The *novel color combinations* category has the same shapes, but the colors are randomly assigned using only those colors that appear in the familiar picture. The *novel shapes* category uses the same layout and the same colors, yet the shapes are randomized. The *novel colors* category uses the same layout and the same shapes as the familiar, but the colors are randomized. The *fully novel* category is different in its layout, and its shapes and colors are randomized. The difference between these stimuli is graded and can be expressed numerically (see details in the text)

The stimulus material was generated with an algorithm using the R language (R Core Team, 2020) with RStudio (RStudio Team, 2016). The algorithm randomly placed five elements in a 3x3 grid. The shape (circle/square/triangle) and the color (blue/green/red/yellow/orange/purple) of the elements were randomly chosen. The algorithm generated a 3x3 matrix containing zeros and ones. The placement of ones represented the placement of shapes. After the matrix representing the layout, the algorithm generated another matrix that represented the shapes at the places defined by the layout matrix. A third matrix represented the color of the shapes. Thus, every picture was represented by three matrices: one for the layout, one for the shapes, one for the colors (see Fig. 1a for an example).

The generation of stimulus material consisted of the following steps:

1. First, the algorithm generated 10 stimuli randomly, avoiding the same layouts. Out of these, the first five were used as *familiars* in the familiarization phase of the experiment (the other five were not used in the experiment, but these were the bases for the *fully novel* stimuli detailed below).
2. Then, using the spatial layout of the stimuli from Step 1, the algorithm generated another four stimuli for all the original layouts whose shape and color were randomly chosen. Importantly, we ensured that these stimuli were not identical to any of the stimuli generated in Step 1. The 20 stimuli corresponding to the five familiars formed the *novel colors and shapes* category, while the 20 stimuli generated by using the layout of the other five original pictures made up the *fully novel* category.
3. Furthermore, the algorithm generated four pictures for each familiar from Step 1 by randomly changing
  - the shapes matrix (*novel shapes;* 5 × 4 = 20) or
  - the colors matrix (*novel colors*; 5 × 4 = 20) or
  - both shapes and colors matrices, but using only colors that were present in the familiar pictures (*novel color combinations*; 5 × 4 = 20).

Thus, the algorithm generated 5 familiars and 100 novel variants (20 in each of the five categories mentioned above; see Fig. 1b for an example). Critically, novels varied in terms of their difference relative to the familiar pictures. From each category, half of the novels were used as novels in the incidental learning phase and the other half were used as distractors in the recognition test phase; thus, the differences were counterbalanced during the recognition memory test. In this study, we used the same stimulus pool for all the participants as the algorithm in its current form produces stimulus pools that differ widely in their internal measurements (i.e. the distribution of difference scores). Studies on category learning indicate that such variability may profoundly influence learning (Sakamoto & Love, 2004); therefore, we decided to use one specific stimulus pool for this study. This decision also reduced the computational needs for the experiment, speeding up the online task (no need to generate a novel stimulus pool for every participant). Supplementary Figs. 7 and 8 show the number of correct responses to each unique stimulus during the test phase (previously presented and distractors, respectively). Based on these figures we believe that the main effects are not attributable to unique stimuli, but appear due to underlying patterns in responding.

To obtain a continuous indicator of novelty, we calculated the difference between novel pictures and familiars. We based these calculations on a method of comparing two pictures. We first compared the layout matrices of the pictures, and any difference between the nine positions resulted in one difference point. For example, if the first layout matrix was [**1,0,**0,1,1,1,0,**0,1**] and the second was [**0,1,**0,1,1,1,0,**1,0**], then the difference would have been 4 (different elements are underscored and highlighted with bold). Then, we compared the shapes matrices of the two pictures with the same method and did the same for the color matrices (these matrices could contain other numeric values, but since these represented categories, the difference was always 1 if there was a mismatch—e. g. the difference between [3,0,0,2,2,2,3,0,0] and [1,0,0,2,2,2,1,0,0] was 2).

### Data analyses and statistics

#### Picture categories

We calculated several difference scores for each novel picture with the previously described method. The difference to the corresponding familiar (DTF) score is calculated between each familiar and the stimuli generated on the basis of its matrices. We also paired every quartet from the *fully novel* category to a familiar randomly, since this category was not based on the familiar stimuli but was generated along with the familiars (as described in 2.2.2. Stimulus material). According to these calculations, the mean DTF for the *novel color combinations* category was 3.4, for the *novel shapes* pictures 3.3, for the *novel colors* category 4.3, for the *novel colors and shapes* pictures 7.1 and for the *fully novel* pictures 16.6. If this metric explains the behavioral performance most closely, then we think it is fair to suggest that novelty detection is based on the comparison of a stimulus to the representation of the stimulus most closely resembling it in memory.

The difference to all familiars (DTAF) score shows the average difference of each novel picture to every familiar. The mean DTAF score was 11.17 for the *novel color combinations* category, 11.71 for the *novel shapes* category, 11.31 for the *novel colors* pictures, 12.35 for the *novel colors and shapes* category and 18.27 for the *fully novel* pictures. If novelty is assessed by the human brain by comparison of a stimulus to several memory representations associated closely with it, then we suggest this metric should be the best predictor of behavioral performance.

Finally, the difference to all novels (DTAN) score is the average difference between each novel picture and all other novels. The mean DTAN score was 13.20 for the *novel color combinations* category, 13.56 for the *novel shapes* category, 13.25 for the *novel colors* pictures, 13.67 for the *novel colors and shapes* category and 17.75 for the *fully novel* pictures. If distinctness within the stimulus set of the recognition test phase drives responses, then this metric should be the best predictor of memory effects.

The calculated difference indices showed significant and strong positive correlations with each other (DTF – DTAF: *r* = 0.88, *n* = 100, *p* <.001; DTF – DTAN: *r* = 0.70, *n* = 100, *p* <.001; DTAF – DTAN: *r* = 0.87, *n* = 100, *p* <.001). These correlations are calculated across stimuli, as the stimulus pool was shared between participants. The distributions of difference scores are visible in Supplementary Figs. 1–5.

#### Incidental learning phase

We used the “afex” package with default settings to conduct analyses of variance (Singmann et al., 2016). We used a one-way analysis of variance (ANOVA) to assess whether there was a difference in the number of correct responses to the novel pictures presented in this phase (factor: PICTURE CATEGORY; levels: *novel color combinations, novel shapes, novel colors, novel colors and shapes, fully novel pictures—ordering was based on the expected variability generated by the stimulus generation algorithm*). We supplemented the ANOVA with a post hoc linear contrast as we expected a gradual decrease in the number of correct responses from *fully novel* to *novel color combinations* pictures (paralleling the mean difference from familiars scores). We also used a mixed regression modeling approach to assess responding to the novels in the study phase. Mixed models are preferred over ANOVA when there are missing values in the data. Furthermore, by taking trial-level data into account, they provide higher statistical power as compared to ANOVAs based on aggregate scores (Lo & Andrews, 2015). Mixed effects modeling also provides a more appropriate way to assess the effect of the calculated difference scores during the recognition test phase. These analyses were also done in RStudio (RStudio Team, 2016), using the lme4 (Bates et al., 2015) and the lmerTest packages (Kuznetsova et al., 2017). In the mixed models of the study phase, the outcome variable was the response given for each novel picture (we coded “old” responses as 0, and “new” responses as 1). The predictor was a difference index (DTF or DTAF). The models included random slope and random intercept for trial type per participant, to account for between-participant differences in response tendency and learning ability, respectively.

#### Recognition test phase

“*Maybe seen*” responses were handled as missed responses in the analyses of the recognition test phase. The prevalence of *“maybe seen”* responses was relatively low in the final sample—mean (± SD): 10.3 ± 6.8). To assess whether demonstrable learning took place during the incidental learning phase, we compared the “*already seen*” responses to old vs. distractor pictures with a Wilcoxon signed-rank test as a Shapiro-Wilk test indicated violation of normality (*p* < .001). Afterwards, we calculated the number of correct recognition responses and subtracted false positives (*corrected recognition score*). We used a one-way ANOVA to examine any differences among the novel picture categories, where ordering was based on the stimulus generation algorithm (*novel color combinations, novel shapes, novel colors, novel colors and shapes, fully novel*). We used one-sample Wilcoxon signed-rank tests to assess which categories were associated with above chance performance.

To further confirm our dose–response hypothesis that more different pictures are recognized with a higher probability, we used mixed effects logistic regression. The models used the test phase response as the outcome variable (coded 1 for *“already seen”* and 0 for *“not seen”*). Predictors were the type of the trial, that is, whether the stimulus in the trial is old (previously seen during the incidental learning phase) or new (distractor), a measure of difference, and their interaction. Given the high correlations between the different indicators of difference, we fitted three separate models (one using DTF, one using DTAF and the last using DTAN as a predictor). The models included random slope and random intercept for trial type per participant, to account for between-participant differences in response tendency and learning ability, respectively.

#### Laboratory experiment

We analyzed the results of the laboratory experiment in the same manner as the online experiment.

## Results

### Incidental learning phase

We observed a 0.399 (± 0.219) mean (± SD) corrected recognition rate for the familiars, well above chance level (chance level would be 0.0, one-sample *t*-test *t*(189) = 25.07, *p* < .001, Cohen’s *d* = 1.82, 95% CI: 1.48 – 2.16). The one-way ANOVA showed a significant effect of picture category (PICTURE CATEGORY: *F*[3.57,335.45] = 24.29, *p* < .001, η^2^G = 0.095). A post hoc linear contrast was significant (*F*(1,94) = −6.74, β = −2.66, *p* < .001), indicating that more novel pictures (i.e. greater difference from familiars) were more likely to be correctly categorized as novel (Supplementary Fig. 6).

The mixed models using difference indices as predictors showed significant main effects. The model using DTF as an index of novelty revealed a main effect of novelty (ß = 0.08, SE = 0.006, *z* = 12.39, *p* < .001). The model on the DTAF score also showed a significant main effect of novelty (ß = 0.05, SE = 0.004, *z* = 12.93, *p* < .001). These results show that the degree of novelty influences responding already in the study phase, and some novel stimuli may not be consistently recognized as such.

We compared the fit of the models with the Akaike Information Criterion (AIC) and the marginal and conditional *R*^2^. The AIC was 7878.8 and 7890.1 for the models using DTF and DTAF as the difference index, respectively. Thus, the model using DTF as an indicator of novelty had the best fit in terms of AIC (AIC difference was 11.3 relative to the model using DTAF). This difference was substantial: according to the criteria proposed by Burnham and Anderson (2004), models that differ from the best-fitting model by at least 10 units of AIC have no support. The marginal *R*^2^ was 0.048 and 0.035, while the conditional *R*^2^ was 0.112 and 0.101, for the DTF and DTAF models, respectively. Thus, entering the difference to the corresponding familiar picture as an indicator of novelty produced the best-fitting logistic mixed model. This suggests that responding during the study phase is most accurately predicted by the model that uses a difference index based on simple comparisons between the actual stimulus and the familiar most closely associated with it.

### Recognition test phase

**Fig. 2:**
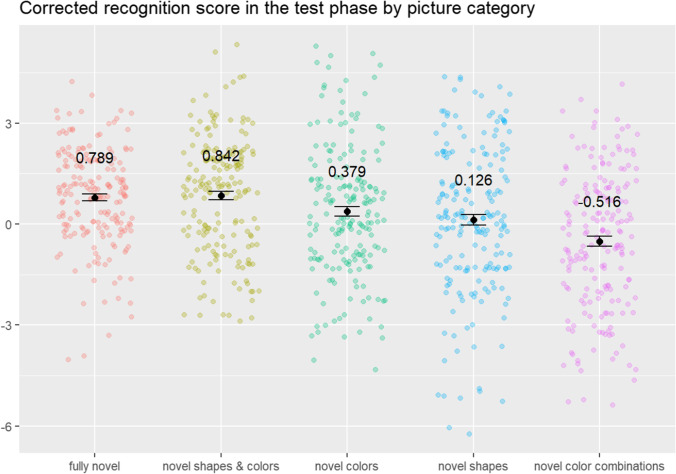
The scatterplot shows the *corrected recognition scores* in the test phase by picture category. The values are close to zero reflecting task difficulty, yet a pattern emerges: corrected recognition is higher for those categories which are more different from the familiars. Colored dots represent individual corrected recognition scores, black dots represent the mean and black bars represent the standard deviation

The Shapiro-Wilk test indicated a violation of normality (p < .001) thus, we used a Wilcoxon signed-rank test, which showed that the responses to the previously presented vs. distractor pictures in the recognition test phase differ significantly (W = 177449, *z* = −5.39, *p* < .001). The one-way ANOVA with PICTURE CATEGORY as a factor to compare the *corrected recognition scores* was significant (PICTURE CATEGORY: *F*[3.78,355.31] = 8.71, *p* < .001, η^2^G = 0. 066), and a post hoc linear contrast showed a significant trend (*F*(1,94) = −6.02, β = −3.33, *p* < .001), indicating that there was a linear relationship among the *corrected recognition scores* to novel picture categories. This indicates that the participants gave more accurate recognition responses to more novel pictures (see Fig. 2). We used one-sample Wilcoxon signed-rank tests to see which categories were associated with above chance performance (corrected recognition scores > 0): for fully novel (W = 8291, *z* = −6.68, *p* < .001), novel shapes and colors (W = 10206, *z* = −6.13, *p* < .001), and novel colors (W = 7257, *z* = −2.37, *p* = .009), we observed above chance performance, while the corrected recognition scores for novel shapes (W = 5851, *z* = −1.27, *p* = .1019) did not differ significantly from chance level. For the category of novel color combinations (W = 4450, *z* = 3.22, *p* = .9994) performance was significantly worse than chance level, meaning that the participants were more likely to give “*old*” responses to distractors from this category.

All mixed models that used a calculated difference index as predictor variable showed significant main effects and interactions. The model with difference from the corresponding familiar (DTF) as an index of novelty revealed a main effect of novelty (ß = −0.14, SE = 0.01, *z* = −16.00, *p* < .001). The trial type (old vs. novel stimuli) also showed a significant main effect (ß = −0.25, SE = 0.09, *z* = −2.86, *p* = .004), and the interaction of these two variables also showed a significant effect (ß = 0.06, SE = 0.01, *z* = 5.59, *p* < .001). The model with DTAF as the difference index showed a significant main effect of the difference between pictures (ß = −0.24, SE = 0.015, *z* = −15.71, *p* < .001) and a significant effect of trial type (ß = −0.93, SE = 0.26, *z* = −3.59, *p* < .001). The interaction of these variables also turned out to be significant (ß = 0.08, SE = 0.02, *z* = 4.22, *p* < .001). Finally, the model with DTAN as the difference index also showed a significant main effect of the difference between pictures (ß = −0.35, SE = 0.02, *z* = −14.98, *p* < .001) and the trial type (ß = −1.38, SE = 0.43, *z* = −3.18, *p* = .001). The interaction was also significant (ß = 0.11, SE = 0.03, *z* = 3.56, *p* < .001).

**Fig. 3:**
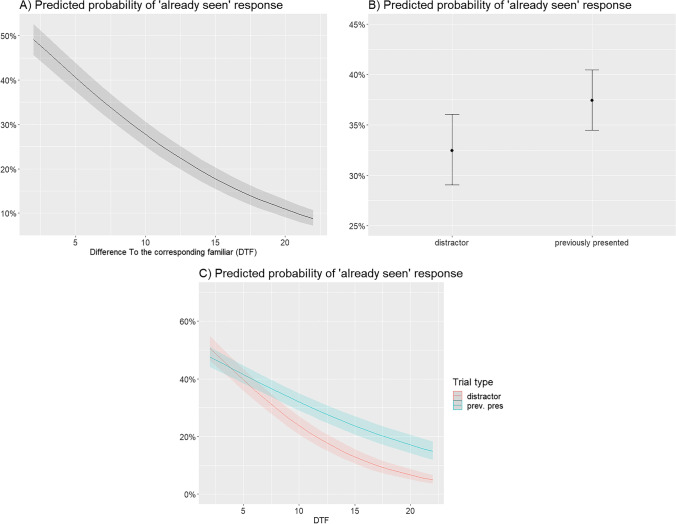
These plots illustrate the main effects and the interaction from the mixed logistic regression model for the probability of an “*already seen*” response during the recognition test phase using difference to corresponding familiar (DTF) as an indicator of novelty. **a** Shows the effect of the difference between the picture and a familiar on the probability of giving an “*already seen*” response on the recognition memory test. The more similar a picture is to the corresponding familiar, the more likely it is to be judged old. **b** Shows the effect of trial type (distractor/previously presented) on the probability of responding with “*already seen.*” Previously presented pictures are more likely to be judged old. **c** Shows the interaction of the difference score and the trial type. Among pictures with a higher value of the difference index (DTF) responding was more differentiated: the ratio of “already seen” responses for previously presented/distractor pictures is relatively increased in this region

These results consistently show that the degree of novelty predicts responses in the recognition test phase. The main effect of the difference scores indicates that the more different a stimulus is, the more likely it is that a participant will respond with “*not seen*” (see Fig. 3a). The main effect of trial type also shows that it was more likely that the participants responded with “*already seen*” to previously presented pictures than to distractor pictures (Fig. 3b). More importantly, the interaction revealed that participants were more likely to give “seen” responses to previously presented pictures that were more novel (i.e. having higher difference scores). In contrast, in the case of pictures with lower novelty (i.e. with lower difference scores), participants were similarly likely to provide “*already seen”* responses to previously seen pictures and to distractors as well. In sum, the higher the novelty was, the higher capacity the participants showed to differentiate previously seen items from distractor ones, indicating improved recognition performance as a function of increasing novelty (Fig. 3c).

Finally, we compared the fit of the models with the Akaike information criterion (AIC) and the marginal and conditional *R*^2^. The AIC was 9289.3, 9306.2 and 9338.2 for the models using DTF, DTAF and DTAN as the difference index, respectively. Thus, the model using DTF as an indicator of novelty had the best fit in terms of AIC (AIC differences were 16.9 and 48.3 relative to the models using DTAF and DTAN, respectively). This difference was substantial based on the abovementioned criteria (Burnham & Anderson, 2004). The marginal *R*^2^ was 0.101, 0.092 and 0.086, while the conditional *R*^2^ was 0.207, 0.198 and 0.191, for the DTF, DTAF and DTAN models, respectively. Thus, entering the difference to the corresponding familiar picture as an indicator of novelty produced the best-fitting logistic mixed model. This implies that the memory effects of novelty might depend on a difference signal that results from the comparison of a novel picture with the representation of a familiar it most closely resembles. The plots showing the predicted probabilities of an “*already seen*” response for the DTF index are shown in Fig. 3.

### Results of the laboratory experiment

The ANOVA on the responses of the incidental learning phase showed a significant effect of picture category (PICTURE CATEGORY: *F*[2.55,45.98] = 5.79, *p* < .003, η2G = 0.079). A post hoc linear contrast was significant (*F*(1,18) = −4.313, β = −2.74, *p* < .001), indicating that more novel pictures (i.e. greater difference from familiars) were more likely to be correctly categorized as novel during the incidental learning phase.

The one-way ANOVA with PICTURE CATEGORY as a factor to compare the corrected recognition scores was significant (PICTURE CATEGORY: *F*[3.22,57.97] = 4.64, *p* = .005, η2G = 0. 164), and a post hoc linear contrast showed a significant trend (*F*(1,18) = −4.60, β = −4.63, *p* < .001), indicating that there was a linear relationship among the corrected recognition scores to novel picture categories.

Next, we used logistic mixed models to assess the data on a trial level in the recognition test phase. We associated each stimulus with three unique difference indices (calculated by comparing the stimulus to a randomly selected familiar, to all familiars [mean] and to all other novels in the same category [mean]). We included the difference index itself as the random slope term in the models for DTF and DTAF and added no random slope term for the model with DTAN. These changes were made in accordance with current guidelines on optimal random effects model structure (Barr et al., 2013). The model with difference from the corresponding familiar (DTF) as an index of novelty revealed a main effect of novelty (ß = −0.16, SE = 0.02, *z* = −6.63, *p* < .001). The trial type (old vs. novel stimuli) also showed a significant main effect (ß = −0.40, SE = 0.19, *z* = −2.11, *p* = .035). The interaction of these two variables also showed a significant effect (ß = 0.09, SE = 0.02, *z* = 3.54, *p* < .001). The AIC of the models using DTF, DTAF and DTAN are 1896.6, 1894.1 and 1917.1, respectively. This differs from the original data, as there is no meaningful difference between DTF and DTAF in this sample.

Overall, the results of the laboratory experiment are in line with the patterns seen in the online sample.

## Discussion

Here, using a novel experimental paradigm, we demonstrate that novelty improves subsequent recognition memory in a dose-dependent manner. We validated our continuous novelty manipulation by showing that the more different a stimulus is from familiarized stimuli, the more likely that it will be distinguished from the familiars during the study phase. Overall, the findings fit with theories of the beneficial effect of novelty on memory and can be interpreted in a predictive processing framework, opening avenues for the application of our paradigm in future neuroimaging and computational experiments.

In this experiment, participants were first familiarized with a set of five pictures. Then, in an incidental learning phase, these familiar pictures were presented several times interspersed with novel pictures, and participants had to judge whether the picture presented was one of the familiarized pictures or a novel one. Participants recognized the familiars above chance level, indicating that they successfully memorized these stimuli. During the recognition test phase, the participants saw the novel pictures from the second phase and the same amount of distractor pictures. Importantly, we quantified the degree of difference from the familiars, providing a straightforward operationalization of the degree of novelty. First, our metric of the novelty of stimuli was dose-dependently related to novelty detection at the behavioral level: we observed a general tendency to respond with *“not seen”* to more novel pictures, regardless of whether they had previously been presented. Second, we confirmed that learning took place during the second phase, as significantly more *“already seen”* responses were given to previously presented pictures. Therefore, we calculated corrected recognition scores and used an ANOVA to test the assumption that novelty (i.e. the degree of difference) influenced recognition memory performance. Importantly, we found that there were more correct recognition responses than false alarms to more different stimuli. Correct recognition responses and false alarms were not different overall for stimuli with lower levels of difference, meaning that the participants did not have distinct memory representations of these stimuli. The number of correct recognitions and false alarms, however, differed for more different stimuli, which could result from the distinct memory representations of these stimuli. Finally, all the key findings were confirmed with a mixed logistic regression model using data from the single trial level, underscoring the robustness of the study. We also conducted a smaller laboratory experiment to verify that in laboratory settings, the dropout rate is sufficiently low. Since most participants had to be excluded due to the high rate of “maybe” responses, we assessed how a more lax criterion (<50 “maybe seen” responses) that retains more participants influenced the results, leaving 105/141 participants in the online sample and 23/24 in the laboratory sample. The analyses on the original and the laboratory data with the lax criterion on “maybe seen” responses were all consistent with the conclusions in the manuscript. Thus, a laboratory environment decreased the dropout rate from 32.6% to 20.8% and to 4.2% with the lax criterion.

We calculated three types of difference indices to reflect novelty. The basis of these difference indices is the calculation described in the Methods section of this paper. The only difference between them is what comparisons are used to calculate the difference index. To calculate the DTF index we compared every novel stimulus to one familiar stimulus. Since this index produced the best-fitting mixed models during both the study and the test phase, we suggest that the encoding of novel stimuli depends on a comparison between the currently seen stimulus and the most similar familiar. If the difference between the novel and the familiar is high enough, a novel memory representation is instantiated. We calculated the DTAF index by comparing novels to all familiars and taking the mean value of these comparisons as the final score. This index was associated with the idea that the encoding of novel stimuli depends on a comparison to all relevant memory representations. This process may be suboptimal relative to the one described above in relation to the DTF index. A single comparison to the most similar representation is probably more computationally efficient than multiple comparisons. Finally, the DTAN index measured the difference of a novel to all other novels. We associated this metric with distinctiveness in relation to other pictures in the test phase. The fact that the model based on this metric produced the worst fit could be taken as an argument against the idea that the distinctiveness of novel pictures is the main effect behind the results presented in this paper, a point to which we will return shortly.

Our results align well with recent ideas on the memory-enhancing effects of novelty, emphasizing that novelty should be considered as a dimension, not a category (Barto et al., 2013; Reichardt et al., 2020; Van Kesteren et al., 2012). However, novelty may be multidimensional. For instance, Duszkiewicz and colleagues suggest that different encoding mechanisms are activated in response to different types of novelty (Duszkiewicz et al., 2019). More specifically, common novelty, which shares some commonality with past experiences, elicits activation in the ventral tegmental area and it facilitates semantic memory formation, while distinct novelty elicits activation in the locus coeruleus, and influences the formation of episodic memories. Relatedly, Kafkas and Montaldi suggest that contextual and absolute novelty elicit different encoding mechanisms (Kafkas & Montaldi, 2018). Contextual novelty detection is accompanied by the activation of the ventral tegmental area and the locus coeruleus, and at the behavioral level, it facilitates recollection. Absolute novelty detection, on the other hand, elicits acetylcholine release into the hippocampus, and behaviorally, it also facilitates recollection. These types of novelty are considered episodic by the authors, in that they lead to better episodic recollection. Finally, the model of van Kesteren and colleagues would suggest that the more different a picture is, the more likely it will be encoded in episodic memory, while similar stimuli may be more likely to enter semantic memory (Van Kesteren et al., 2012). Although this model concerns itself with high-level knowledge, the main assumptions could be extended to include any type of prior knowledge that could influence the encoding of related stimuli. Since statistical regularities can be considered a form of prior knowledge, the visual patterns of the familiar stimuli could be expected to affect encoding not only when these patterns are highly different, but when they are highly similar too. Our results, however, do not show a consistent memory effect for highly similar stimuli.

In our experiment, it would seem straightforward to categorize all stimuli as showing degrees of common novelty as Duszkiewicz and colleagues suggest (Van Kesteren et al., 2012) or contextual novelty as Kafkas and Montaldi propose (Kafkas & Montaldi, 2018), as the stimuli are highly similar. However, novelty should be defined relative to a context: we cannot declare the novelty of a stimulus by judging only the stimulus itself; we must consider the whole stimulus pool (Rust & Mehrpour, 2020). In our experiment, the stimulus pool consisted of very similar stimuli, among which some were more different from the familiarized stimuli than others. In this stimulus pool, a distinctly novel stimulus may be very similar to other stimuli, yet these are the most outstanding considering the details of the pictures. This aligns well with the predictive interactive multiple memory systems (PIMMS) model of Henson and Gagnepain (Henson & Gagnepain, 2010). According to the PIMMS model, when the observers’ expectations (prior) differ from the actual input (likelihood), then a prediction error is generated which acts as a learning signal to refine the predictions. In this task, the familiarization phase produces strong expectations for the presented stimuli and the pictures seen during the study phase generate prediction errors which scale with the difference from the familiars. Our results support this interpretation, even though the expectation or the prior must also be shaped by the trials in the study phase. The extended version of the PIMMS model also suggests that the size of the prediction error has a U-shaped relation to learning (Quent et al., 2021; Van Kesteren et al., 2012). This would mean that those stimuli that differ minimally from previously encountered stimuli are also more likely to be encoded. We could not demonstrate this effect in our study, probably because the stimuli that were highly similar to the familiars were also very similar to each other. A more carefully balanced stimulus pool may reveal the full spectrum of the memory effect of the degree of novelty.

Of note, the task in its current form is not amenable to distinguish between the notions currently used to describe memory formation. Unique memory effects resulting from novel, surprising, unexpected, deviant or distinctive information are not trivial to distinguish (Barto et al., 2013; Reichardt et al., 2020; Schomaker & Meeter, 2015). The task emphasizes the differences between the stimuli which we called novelty throughout this paper, but it can also be conceptualized as the degree of unexpectedness. In this task, novelty and unexpectedness coincide, as expectations for the visual stimuli probably form in a way that familiar stimuli become somewhat expected but different stimuli less so. Yet, we think that generating stimuli that vary in a continuous manner could supplement already existing methods for manipulating expectations. The degree of novelty could be defined as the degree of difference from previously presented stimuli and the degree of unexpectedness could be calculated as the degree of difference from the expected stimuli. These two could be manipulated separately to see how they influence memory. The importance of this opportunity is that novelty and unexpectedness are not consistently distinguished in the field, although the relevance of this distinction has been pointed out in the past (Barto et al., 2013). The most novel or most unexpected stimuli can also be deemed deviant, distinct or surprising, adding to the conundrum.

The Graded Novelty Encoding Task we developed is unique in its continuous operationalization of novelty. To our knowledge, previous studies have only differentiated between two levels of novelty, at best. For example, Greve and colleagues manipulated the degree of novelty in a task employing picture-emotional word associations by changing the word to one of similar meaning or to one with an entirely different meaning (Greve et al., 2017). This approach yielded trials with low and high novelty, respectively, and showed a recognition memory benefit for high novelty trials. A similar approach has been used in paired-associates tasks, where the degree of novelty was manipulated by rearranging word pairs or showing a familiar word in the presence of an entirely novel one (de Chastelaine et al., 2017). Finally, by using sequences of images, Kumaran and Maguire also manipulated the degree of novelty in several of their experiments (Kumaran & Maguire, 2006, 2007, 2009). In these studies, participants were familiarized with series of pictures and later tests mixed the sequences or even switched some pictures yielding low and high novelty trials. We believe however, that with the method presented in this paper, researchers will be able to better control the degree of novelty and to assess the processes involved in novelty detection and memory encoding more efficiently and in greater depth.

The parametric manipulation of stimuli can also be useful in the mnemonic similarity literature. In the Mnemonic Similarity Task (MST), participants are asked to recognize pictures that are similar to those that they learned during a previous task phase (Stark et al., 2013; Yassa & Stark, 2011). Their responses to these pictures (so-called lures) can be used to make inferences about the precision of their memory representations of the original picture. The similarity of the stimuli is mostly inferred from the responses of the participants, but there have been studies with parametrized stimuli. For example, some researchers have used morphed faces, where the similarity of a face could be expressed with a percentage to a familiar face (Bencze et al., 2021; Chang et al., 2015). In another study, the degree of rotation was changed to manipulate similarity parametrically (Motley & Kirwan, 2012). The stimulus pool of the Graded Novelty Encoding Task can also be parametrically manipulated, along more dimensions, which could be an interesting addition to the MST. Adding a numerical value describing the degree of difference between an originally presented picture and a lure, researchers could calculate the threshold which determines whether pattern completion or separation takes place upon seeing a stimulus that is similar to a memory representation.

Some crucial points in relation to the notion of “distinctiveness” remain to be answered. The first one is related to the von Restorff effect, which shows that distinctive stimuli are recognized with a greater probability (Hunt, 1995). Distinctiveness is a closely related notion to novelty, but novelty is a more general term describing anything that is not contained in the memory system, while distinctiveness presupposes that the stimulus in question is not only novel, but it also differs profoundly from anything represented in memory. The memory effects of distinctiveness have been theorized to originate from attentional processes during encoding (Schmidt, 1991), but later it turned out that the effect may be instantiated during retrieval itself (Hunt, 1995). The general idea is that the similar stimuli presented during the experiment get agglutinated in memory, while the distinct stimulus remains distinct in memory making the representation easily accessible resulting in better recognition performance. Our task in its current form is not able to add a novel argument to this debate. However, we believe that the distinction between distinctiveness and novelty in experimental psychology may be misguided (Reichardt et al., 2020). Distinctiveness, or in other words the more extreme values of novelty, could be the only relevant values of novelty when it comes to paradigms with hundreds of similar stimuli. The strength of this paradigm lies with the fact that the differences between stimuli can be manipulated and distinctiveness can be more precisely defined. The paradigm in its current form shows that novelty itself is not necessarily meaningful for the observer, but probably only from a certain threshold, which can be called the threshold of distinctiveness: a degree of difference that requires a distinct engram to form. Neuroimaging studies using the GNET may shed new light on the differences between the neural processes taking place at encoding and at retrieval.

The second question related to the experimental tasks assessing the memory effects of novelty focuses on the recognition memory test. Responses to distractor stimuli in the test phase are used to calculate the so-called false alarm rate, which shows the reliability of the correct responses during the test. A classic study showed that the corrected hit rate for novel items on word lists is higher than the corrected hit rates for familiar words, which was attributed to the memory effect of novelty (Tulving & Kroll, 1995). However, the difference between corrected hit rates for the different categories seems to originate from a difference between false alarm rates, while the hit rates were equal (Dobbins et al., 1998). Therefore, source confusion among the familiar words seems to be a more likely explanation. Relatedly, in our experiment some of the novel pictures were very similar to the familiars. In fact, the main effect of the difference indices in the mixed models of the test phase data is clearly attributable to source confusion: less-different novels could be mistaken for the familiars and thus the difference indices are inversely related to the proportion of recognition judgements, so correct recognitions and false alarms decrease in parallel as the difference indices increase. Yet, the interaction term in the models show that the proportion of correct recognitions and false alarms differ significantly at higher difference indices. We related this finding to better recognition of previously presented highly different stimuli, but stating that the effect stems from better detection of highly different novel stimuli may be just as valid. These two explanations are the two sides of the same coin here: hit rate and false alarm rate decrease together, while the number of misses and correct rejections increase in parallel. Since using distractors during a recognition memory test is necessary to assess the reliability of responding, this problem is inherent to paradigms using a recognition memory test. Even though the Graded Novelty Encoding Task in its current form is not able to overcome this problem, the algorithmically generated stimulus pool may be used in other setups to avoid it. For example, as the stimuli are made up of simple, colored shapes, a recall test may also be feasible which eliminates the requirement for distractor stimuli during testing.

Some methodological issues should also be highlighted. First, we used an additional response option in the recognition test phase and later excluded these responses from the analyses. In a future study, it may be more practical to use only two response options or collect confidence ratings. Another caveat is the relatively low performance of the participants which is probably due to the difficulty of the task. At the same time, it should be noted that performance was not at floor and as shown by our models, there was meaningful variation in task performance. The calculation of differences is also a point where strides could be made to more precisely differentiate between the stimuli. A more controlled approach to the generation of the stimulus pool could help in differentiating between the impact of the dimensions of the stimuli, as there are reasons to think that shapes and color, for example differ in their impact on memory (Allen et al., 2006). Future studies could apply manipulations to uncover the effects of encoding strategies, for instance, by including a deep encoding (e.g. “Is there a red triangle in the picture?”) or an intentional encoding condition. The use of a colorblind-friendly color palette could expand the eligible circle of participants. We hope that the Graded Novelty Encoding Task will be a useful addition to the methodological palette of memory research to further understand the rules of memory formation. We believe that using stimuli that are quantifiable is an important step forward, as the usage of verbal categories often leads to conflicting results that are hardly reconcilable (Barto et al., 2013; Reichardt et al., 2020; Schomaker & Meeter, 2015).

In sum, the Graded Novelty Encoding Task is an exciting new paradigm that manipulates the differences between the presented stimuli parametrically. The first result with the task lends support to the idea that the more different a stimulus is from previously encountered stimuli, the more likely it is that it will be memorized. This basic assumption is in line with current thinking about the functioning of memory (Frank & Kafkas, 2021; Quent et al., 2021). The main motivation of our article is to show that expressing the degree of novelty as a quantity is more meaningful than using categorical descriptions like stimulus novelty, oddball, incongruent stimulus or spatial novelty. The Graded Novelty Encoding Task will hopefully be an important addition to the toolbox of memory researchers.

## Supplementary Information

ESM 1 (DOCX 244 kb)
