# Supplementary material for: The graded novelty encoding task: Novelty gradually improves recognition of visual stimuli under incidental learning conditions
Source: Behav Res Methods. 2022 Jun 13;55(4):1587–600. doi: 10.3758/s13428-022-01891-8 (PMC10250520; doi:10.3758/s13428-022-01891-8)
Supplement: Supplementary file 1 — (DOCX 244 kb) [file 13428_2022_1891_MOESM1_ESM.docx]

# Supplementary Material


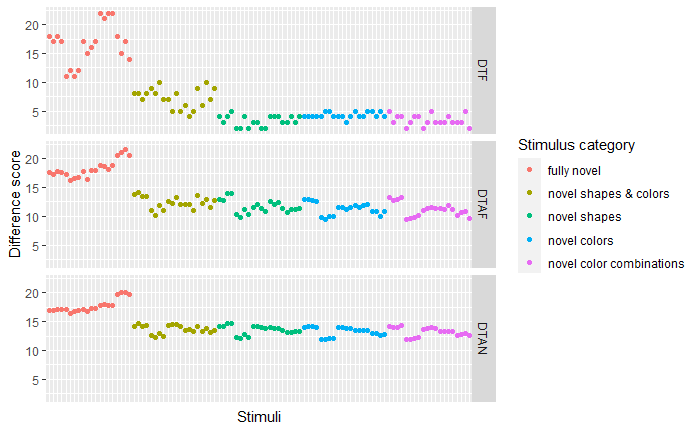


Supplementary Figure 1. – The values of the three difference scores in the stimulus set used in the experiment. The values are calculated by comparing the three matrices describing a picture to the matrices describing another picture or pictures (see “Picture categories” subsection of the “Data analyses and statistics” section). DTF is the difference to the corresponding familiar, DTAF is the mean difference to all familiars and DTAN is the mean difference to all other novels. Calculation of these difference indices is detailed in the text.


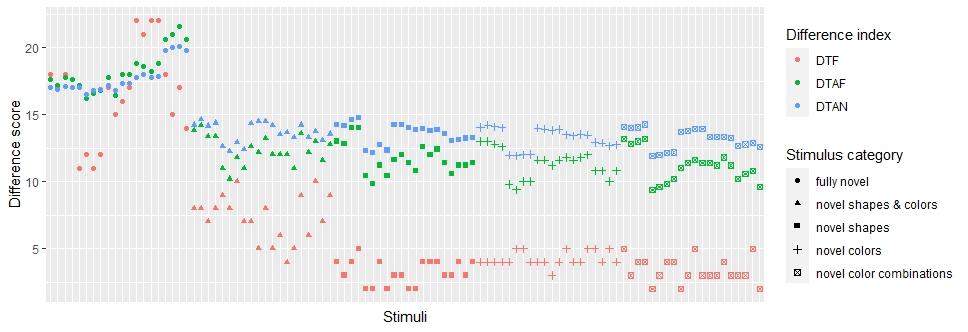


Supplementary Figure 2. – The values of the three difference scores in the stimulus set used in the experiment, visualized in a single space. The values are calculated by comparing the three matrices describing a picture to the matrices describing another picture or pictures (see “Picture categories” subsection of the “Data analyses and statistics” section). DTF is the difference to the corresponding familiar, DTAF is the mean difference to all familiars and DTAN is the mean difference to all other novels. Calculation of these difference indices is detailed in the text.


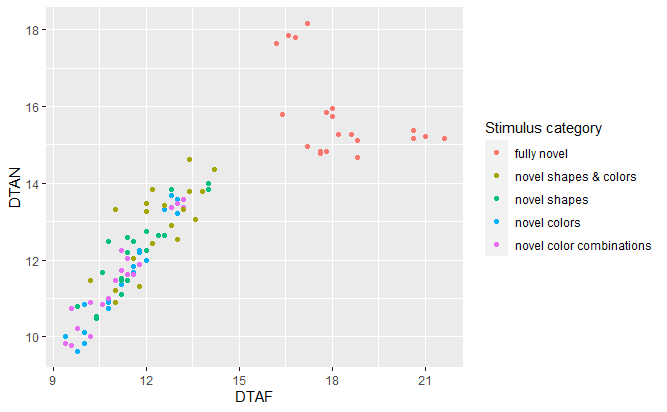


Supplementary Figure 3. – This figure shows the pairwise association between the difference indices DTF and DTAF.


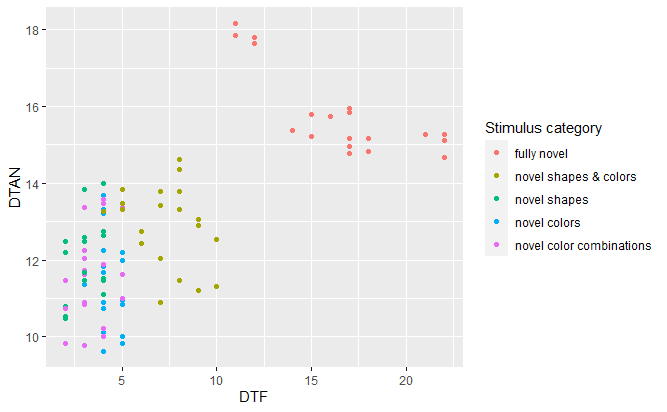


Supplementary Figure 4. – This figure shows the pairwise association between the difference indices DTF and DTAF.


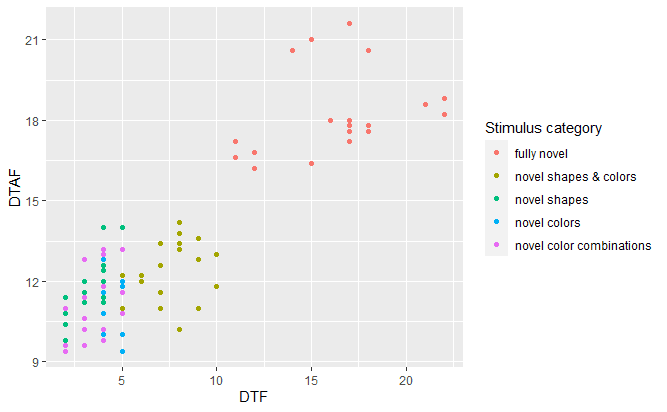


Supplementary Figure 5. – This figure shows the pairwise association between the difference indices DTAF and DTAN.


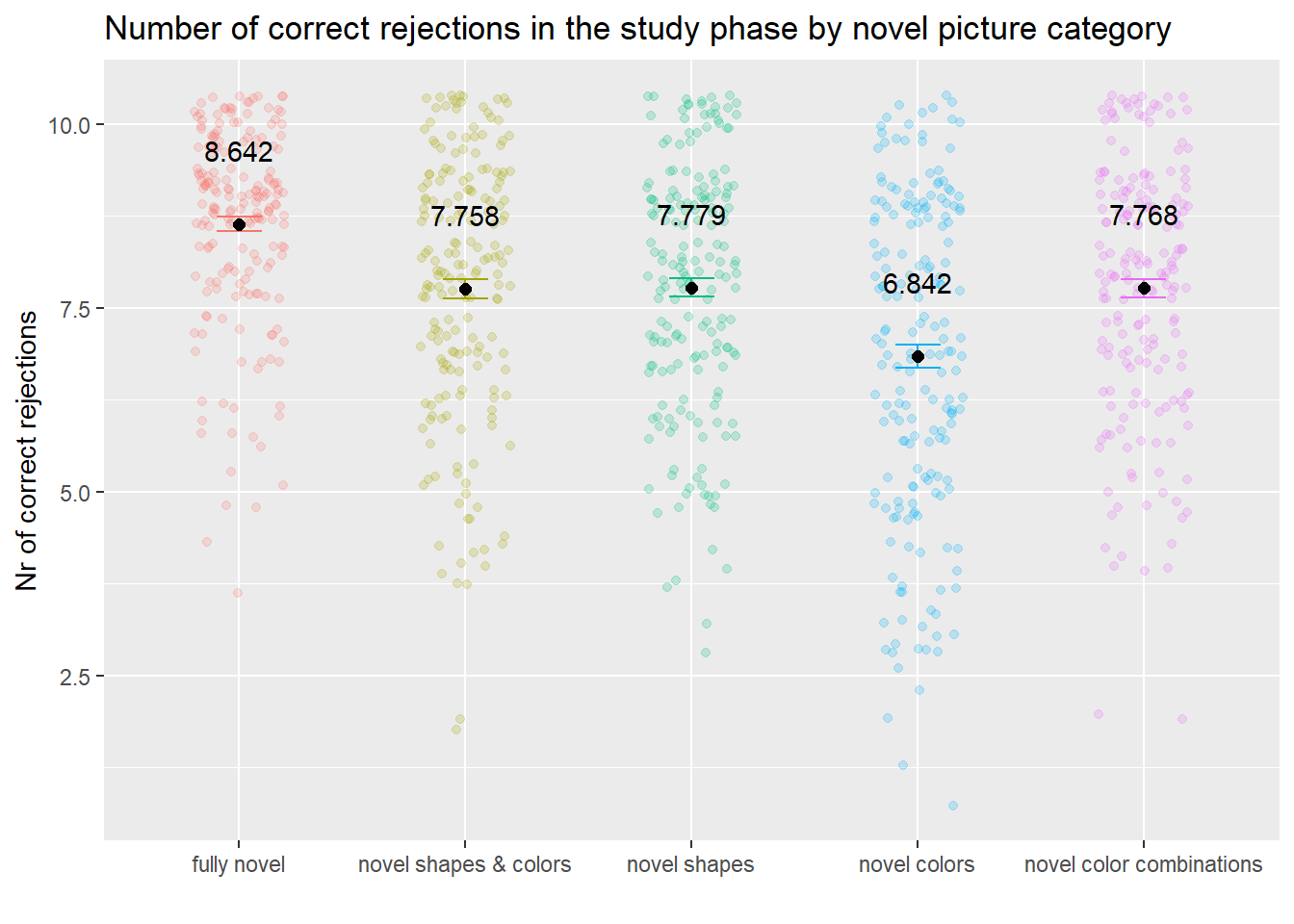


Supplementary Figure 6. – Number of correct rejections in the study phase by novel picture category. The participants had to decide upon seeing a picture if it is one of the familiars or a novel picture. Every novel picture category was represented by 10 pictures in this phase. Dots show the mean and error bars indicate the standard error.

*
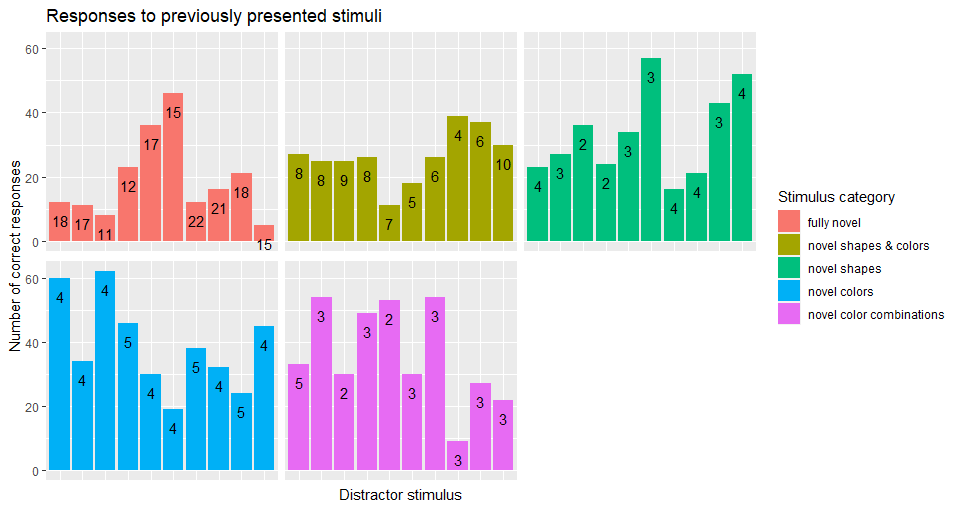
*

Supplementary Figure 7. – Responses to previously presented stimuli during the test phase. Columns represent the number of correct (‘*already seen*’) responses to each stimulus in the category. Numbers in black represent DTF score. The highest score in the fully novel category (47) corresponds to about half of the participants (N=95).

*
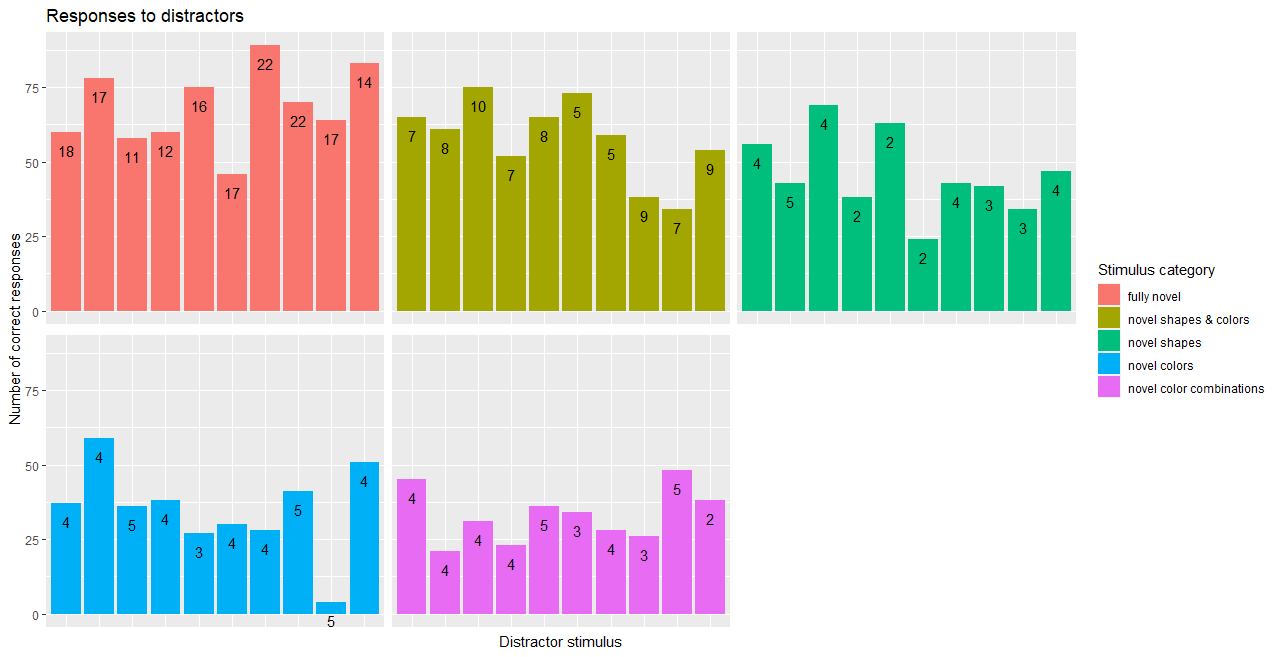
*

Supplementary Figure 8. – Responses to distractor stimuli during the test phase. Columns represent the number of correct (*‘not seen’*) responses to each stimulus in the category. Numbers in black represent DTF score.
